# Determinants and Prevention Strategies for Household Food Waste: An Exploratory Study in Taiwan

**DOI:** 10.3390/foods10102331

**Published:** 2021-09-30

**Authors:** Chih-Ching Teng, Chueh Chih, Wen-Ju Yang, Chia-Hui Chien

**Affiliations:** 1 Department of Restaurant, Hotel, and Institutional Management, College of Human Ecology, Fu Jen Catholic University, New Taipei City 242062, Taiwan; 2 Graduate Institute of Sport, Leisure and Hospitality Management, National Taiwan Normal University, Taipei City 10610, Taiwan; 80831001a@ntnu.edu.tw; 3 Department of Hospitality Management, Hungkuo Delin University of Technology, New Taipei City 236302, Taiwan; iwenju@gmail.com; 4 Department of Food and Beverage Management, Lee-Ming Institute of Technology, New Taipei City 24352, Taiwan; anne@mail.lit.edu.tw

**Keywords:** household food waste, food waste reduction, food knowledge and skill, food waste prevention strategy, household food provider

## Abstract

Given the large amount of food waste coming from households, reducing household food waste is essential to the mitigation of overall food waste and the provision of multi-faceted benefits for both people and the planet. This study identifies factors and management strategies for the reduction of household food waste in the Taiwanese household setting. Using snowball sampling, semi-structured interviews are conducted to collect data from 27 household food providers in Taipei. The research findings identify four critical motivators and four barriers to minimizing household food waste in Taiwan. The most frequently mentioned motivator for the reduction of food waste is a convenient shopping environment, and the most important barrier is lack of knowledge for assessing the edibility of food. Additionally, four major prevention strategies are identified to help reduce household food waste: (1) planned purchase schedule; (2) skills to keep food fresh and longer; (3) understanding family preferences and leftover management, and (4) sharing additional food and co-procurement and cooking. The results of this study not only help improve the understanding and application of Chinese household food waste reduction, but also demonstrate the significance of its socio-cultural impacts in future studies.

## 1. Introduction

The UNEP food waste index report (2021) estimates that food waste from households, retail establishments and the food service industry totals 931 million tons each year. The proportion of global greenhouse gas emissions from food that is not consumed is estimated as 8–10% [1]. According to the statistics of the Waste and Resources Action Programme (WRAP) (2020), 70% of food waste in the UK in 2018 came from households where two-thirds of the food waste was edible [2]. Considering the large amount of food waste coming from households, lowering household food waste is essential to mitigating overall food waste and providing multi-faceted benefits for both people and the planet. Previous studies have identified factors associated with household food waste. Aschemann-Witzel et al. (2015) found that consumers’ motives to avoid food waste and their food management skills significantly affect household food waste behavior [3]. Mattar et al. (2018) further indicated that cultural and socio-economic backgrounds influence household consumers’ food consumption and waste behavior [4]. To respond to these issues, researchers need to investigate factors affecting household food waste from different regions and cultural backgrounds to provide appropriate strategies for reducing household food waste.

Located in Eastern Asia, Taiwan has a total population of 23 million. Although Taipei City covers only 6% of the total area of the main island of Taiwan, it accommodates about 30% of the total population, while contributing more than 30% of the total kitchen waste [5]. Therefore, to reduce food waste in Taiwan, priority should be given to minimizing household food waste in Taipei. Although previous studies have explored the motives of household food waste [6,7] and adopted the Theory of Planned Behavior (TPB) to examine variables influencing household food waste behavior [8,9], most were conducted in the West and rarely surveyed consumers in Asian countries and Taiwan.

Taiwan has its own specific cultural and geographical characteristics distinct from western countries, and these affect food purchase and consumption in Taiwanese households. For example, Taiwan has quite diverse and convenient food-purchasing channels, including traditional markets, mass merchandising supermarkets and retail stores. In particular, the density of convenience stores is extremely high with an average of one convenience store per 2148 people to provide services nearby [10], selling various heated and ready-to-eat goods. Some stores even sell fresh vegetables and fruits delivered directly from farms. Previous works have found that high-density and convenient food retail infrastructures can affect the habit and quantity of household food purchases [11]. In socio-cultural terms, Taiwan inherits the Chinese culture where people use gift-giving as a tool for emotional connection and market exchange. When gift-giving involves symbols of power and status, the gift-givers generally give more food than the recipients need [12,13]. Food gifts from relatives and friends may also cause hidden worries of household food waste. Moreover, Taiwan’s densely populated geographical environment promotes vibrant regional interpersonal interaction. The food-sharing network built by community interpersonal relationships enable timely and efficient sharing of food purchases and other food redistribution.

As noted above, factors affecting household food waste behavior found in previous studies may vary by region and socio-cultural background. Given that few studies have explored household food waste behavior in Taiwan, researchers have difficulty in understanding the determinants of household food waste behavior for developing prevention strategies. Therefore, the current study investigates motivators and barriers to minimizing household food waste, and identifies strategies for preventing household food waste in Taiwan through a qualitative approach. The results of this study will hopefully provide deeper understanding and make valuable suggestions for the government and policy-makers to decrease household food waste, particularly in the context of Taiwanese households.

## 2. Literature Review

Studies on household food waste over the last two decades have mainly focused on understanding who are most likely to throw food away and how people feel toward food waste [14]. Although these studies provide an in-depth analysis of the subject groups, they cannot explain the causes of household food waste. Evans (2011) conducted a sociological study on the eating habits of 19 British households and identified the potential issues on how and why households throw food away, providing useful factors for further examination on reducing household food waste [15]. Through semi-structured interviews with 15 British households, Graham-Rowe, Jessop, and Sparks (2014) investigated the feelings and experiences of the household food purchasers and found that the two main factors for household food waste reduction were waste concerns and doing the “right” thing [6]. Their study also emphasized that the significance of food-management skills in reducing food waste and identified factors that hinder the reduction of food waste, such as reducing inconvenience in life, lack of priority and avoiding responsibilities. Additionally, the Theory of Planned Behavior (TPB) is widely adopted to examine the relationships between variables affecting household food waste behavior, and has identified variables such as attitude, subjective norm and perceived behavioral control as significant predictors [8,9,16,17,18].

The current study analyzed and categorized related literature to understand relevant factors and management strategies influencing household food waste behavior prior to performing the semi-structured interviews. Table 1 presents a list of studies that have identified antecedents of household food waste and management strategies to reduce food waste. The antecedents of household food waste include factors that promote and inhibit waste reduction. Motivations to minimizing household food waste include awareness, norm, attitude, behavioral control, habit, and health perception. Awareness refers to the environmental and waste concerns of consumers [6,19]. Previous works have suggested that environmental concern is the second most important facilitators [20]—next to financial concern—for households that determine their willingness to reduce food waste [6]. Meanwhile, norms include subjective norms [17], social injunctive norms [18], moral norms [18] and personal norms [21]. However, many previous studies have suggested that the connection between norms and actual behaviors is weak, since household food waste is practiced in private and is not easily seen by others. Therefore, in the absence of public scrutiny, the influence of morality on behaviors is weakened [22]. Additionally, factors relevant to personal attitude are also common motivators affecting households in reducing food waste, such as moral attitude, attitude towards food waste and anticipated regret [8,9]. Finally, factors relevant to behaviors include perceived behavioral control, habitual food waste behavior and perceived health risk, among which the perceived behavioral control [8,17] and habitual food waste behavior [9] have significant effects on a household’s food waste behavior. Furthermore, food waste increases as consumers become more perceptive of health risks [21] and of their choices over safe and healthy food [23].

Role identity, eating preferences and lifestyle were identified as impediments to food waste reduction in previous studies. Role identity refers to the degree to which an individual considers a particular role to be part of oneself [24]. Two common role identities of household food providers are good provider identity [6,21] and self-identity [8]. In particular, good provider identity maintains that a provider can qualify for the role of “good food provider” only by providing large amounts of food. Consequently, providers tend to provide large amounts of food to meet the needs of their family members [21]. Conversely, consumers with pro-environmental self-identity have a stronger motive to reduce household food waste [8]. Other behavioral characteristics related to family members, such as unpredictable eating behaviors and preferences [25] are also mentioned in prior studies. Due to unplanned events (e.g., a spontaneous decision to have take-outs or go out for dinner, or when family members cannot come home for dinner at short notice), the original cooking plan might be canceled or changed, which will lead to the deterioration of excess raw and fresh food materials in meal preparation [22,26,27]. Additionally, household food providers who are under time pressure may choose to purchase and stock food in large quantities in order to minimize the inconvenience [6]. The over-preparation of these stockpiled foods and leftover food will cause doubts over food safety and family members would feel disgusted or sacrificed with leftovers [3,25,28]. All these factors would result in an increase in household food waste.

Food consumption management and skills play critical roles [22,29] and significantly lower the amount of household food waste [29]. Common household food management problems include inappropriate storage, lack of food provision and poor stock planning [30], poor meal planning, improper cooking and cooking too much [31]. Both Stefan et al. (2013) and Stancu, Haugaard, and Lähteenmäki (2016) found that shopping and planning routines directly affect the degree of household food waste [17,18]. Previous studies have identified the stages of food management strategies as food procurement, storage, preparation, supply, consumption and waste disposal [29]. Consumers adopt various methods to assess whether food is edible and safe to be eaten [32], including assessment by smell or shelf life of food [20,32]. With the increase in food disposal due to concern over food safety [32], appropriate edibility assessment, knowledge of food shelf life and how to extend it, and food storage methods are necessary and effective strategies in reducing household food waste.

Although recent literature has discussed factors related to household food waste in western countries [22,30], few studies have addressed the issues in the context of Asian households. Schanes et al. (2018) stressed that social, economic, and cultural structures will affect the strategies that can be adopted by individuals to prevent food waste in their households. As a household is embedded in the broader social, economic and cultural structure, it is necessary to re-examine antecedents and behaviors of household food waste under different cultures.

## 3. Method

The current study adopted a qualitative approach to investigate perceptions and experiences of household food providers regarding household food handling and practices. Individual, semi-structured, in-depth interviews were used to collect data, as this method enables the researchers to elicit perceptions and opinions from the respondents. Before conducting the interview, all participants were informed the purpose of this study and the interview procedure and guidelines, and were assured that their personal information would not be disclosed, transmitted or distributed. Additionally, the participants must consent to participate in this study voluntarily. The interview results were ensured not list any identifiable information and only limited for the use of academic research.

### 3.1. Participants

This study adopted a combination of purposeful and snowball sampling to select respondents who could articulate their thoughts and opinions to improve understanding of household food handling, food waste production and food waste prevention strategies. To be eligible for the study participants, the initial respondents had to meet the following selection criteria: (a) 18 years or older, (b) Taiwanese resident, and (c) household food providers responsible for food purchasing and cooking. Interviewees were contacted through personal contacts of the researchers, and via subsequent snowball sampling. Regarding snowball sampling, after completing each interview, respondents were asked to recommend other potential participants who also meet the above selection criteria. Accordingly, 27 household food providers in Taipei were selected as in-depth interviewees. Among the respondents, 17 lived in Taipei City while 10 lived in New Taipei City. Nearly 80% of the respondent’s households comprised a husband, a wife and either one child or two children, which is consistent with the general household structure of Taiwan [34]. Most of the respondents were 30–50 years old. Only two of them were males while the rest were females. Full-time housewives accounted for 30% of the women, whereas professional women accounted for 70%. Additionally, most respondents were educated to college level or above. Table 2 shows the profiles of the respondents.

### 3.2. Procedure and Data Analysis

This study performed individual pilot interviews to develop the main interview guides before the main interviews. The initial interview guides were developed based on the literature related to household food waste. Three pilot interviews were conducted to ensure the appropriateness of the interview questions. Based on the results of the pilot interviews, several questions related to cooking, food preparation, and food management were included in the main interview guides. Finally, the interview format incorporated five sections, with questions on perceptions and experiences about food purchasing, food storage, food preparation and cooking, waste behaviors and methods and strategies for the reduction of food waste. To ensure that all the key factors that influenced household food waste were discussed, the interview included questions on factors that drive and hinder the reduction of household food waste, and strategies to prevent household food waste. Table 3 shows the final interview questions and their links to the extant literature.

During the data collection, one of the researchers, who was experienced in performing in-depth interviews, conducted all the interviews. Each interview lasted 90 min on average, and was recorded and transcribed verbatim for data analysis. After data collection, the researchers used content analysis to code and categorize the interview data and sorted out their frequency distribution according to their characteristics via systematic data comparisons [35]. Reliability testing was undertaken using Holsti’s method (1969) [36]. The reliability index, based on the replicability and correctness of the results from two coders, was more than 0.80, indicating that the coders had a high degree of mutual agreement on the categories of analysis units. In terms of validity, two experts familiar with qualitative research were invited to examine the content validity of the data analysis. Both experts agreed with the categorized results found by the researchers, indicating that the analytical results have an appropriate degree of content validity. Additionally, this study performed data triangulation, including reviewing the literature, reflecting on interview notes, and cross-examining the data from both the interviewees and the researchers, confirming the validity of the study.

## 4. Results and Discussion

**Figure 1:**
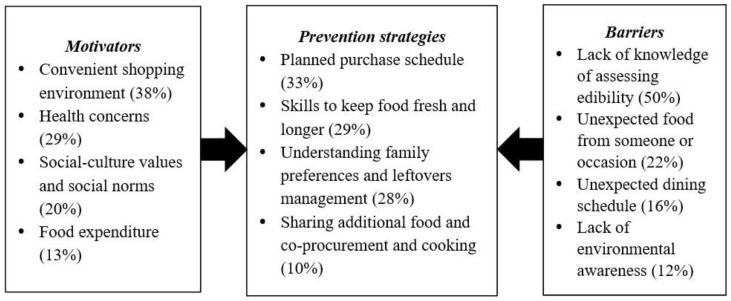
Determinants and strategies for household food waste reduction. Note: The number in parentheses denotes the percentage of frequency obtained from content analysis.

Figure 1 presents the analytical results, which identify the motivators, barriers and prevention strategies related to the reduction of household food waste. The four core categories of motivators to reduce household food waste, in order of frequency based on content analysis, are (1) convenient shopping environment (38%), (2) health concerns (29%), (3) social-culture values and social norms (20%), and (4) food expenditure (13%). The analysis also identifies four core categories of barriers to minimizing food waste: (1) lack of knowledge of assessing edibility (50%), (2) unexpected food from someone (22%), (3) unexpected dining schedule (16%) and (4) lack of environmental awareness (12%). Four food waste prevention strategies are (1) planned purchase schedules (33%), (2) skills to keep food fresh and longer (29%), (3) understanding family preferences and leftover management (28%), and (4) sharing additional food and co-procurement and cooking (10%).

### 4.1. Motivators to Reduce Food Waste

#### 4.1.1. Convenient Shopping Environment (38%)

The food supply system plays a significant role which can either restrict or promote consumers’ consumption behaviors and food practices [37]. The reasons behind the convenience of Taiwan’s food supply system come from three conditions: high-density convenience stores, emerging online shopping and living near one’s friends and relatives. In particular, Taiwan has the second highest density of convenience stores in the world. Conversely, the emergence of the Internet provides convenient shopping channels for households. Although online purchasing channels for fresh food are not entirely common in Taiwan, individual and group-buying businesses in social media and communities are quite active. Interview results demonstrate that the household purchase channel for food has gradually changed from a single traditional market to a multi-channel purchase. This development not only increases the frequency of food purchases but also reduces the habit of storing food, and thus plays a positive role in decreasing household food waste. Other studies have found that in South Korea, which has a similar convenient shopping environment to Taiwan, a household that buys fresh fruits and vegetables every day can reduce its avoidable food waste by 54% when compared with those that buy fresh fruits and vegetables two or three times a week, and by 69% compared to those that buy once a week [37]. Therefore, the accessibility and density of food retail infrastructures shapes household food purchase habits, further affecting the amount of household food purchases [11]. Several interviewees stated:

*“Now I’m used to buying vegetables online…All the goods will be delivered to home. For example, the delivery man can buy what I need in an organic store, he can also go to the Binjiang Market to buy vegetables and fruits that I need, and then deliver to my home all together. It is very convenient and free from impulse purchase.”*

(A_11)

*“When food is not enough, I go to the PX Mart chain. Because it’s so convenient in Taiwan, with three convenience stores or supermarkets just around the corner, I always feel that there is no need to stock up on food.”*

(A_7)

#### 4.1.2. Health Concerns (29%)

This study found that respondent householders who are more concerned about health and food safety exhibit more conscious behaviors in food selection and purchase, such as choosing organic stores or purchasing food through specific channels, and pay more attention to the impact of food on the environment and resources [38]. Therefore, the interview respondents’ main motives in food selection and purchase were food quality, health and safety, and environmental concerns, rather than food prices. The analytical findings are consistent with Quested et al. (2013), which indicate that a healthy diet can encourage consumers to inspect the nutrient labels and shelf-life more frequently [19], and consequently lower a household’s food waste [32]. Additionally, some respondents emphasized that from the marketing perspective, products that are advertised as healthy and safe are usually expensive. Graham-Rowe et al. (2014) define healthy and safe foods as foods that can protect and nurture human health, and will not cause foodborne illness (e.g., food poisoning) [6]. To make “healthy and safe” food affordable for ordinary households, these products are typically sold in small packages in Taiwan. This phenomenon also indirectly appeals to health-inclined household consumers to avoid buying food products in large packages or quantities, in turn reducing food waste.

*“… Organic or safe food products with good quality usually come in small packages, it is fresher and easier to keep, and unlikely to have leftovers.”*

(A_8)

#### 4.1.3. Socio-Cultural Values and Social Norms (20%)

Crops are traditionally a symbol of wealth for households deeply affected by their socio-cultural background and agriculture-based economy. In Taiwan, food is highly respected and must not be wasted easily. Although Taiwan’s economic development has switched its focus from agriculture to industrial and commercial services, the government has frequently released policy initiatives to attract young people in developing new agriculture in rural areas, and rethink issues on food waste and the environment. Traditional cultural values and new agriculture policies have been found to affect young people’s eating attitudes. These values and norms over food consumption are shown in the following respondent’s response:

*“Every grain of rice is from hard work. I have known it since my childhood. I feel guilty towards the farmers if I do not finish my rice. Well, my grandparents were self-employed farmers, so I am especially concerned about it.”*

(A_11)

This study also found that the feeling of guilt is an important psychological motive that influences food waste reduction. Respondents who associate their food waste behaviors with feelings of guilt are more likely to take actions in reducing food waste. Consistent with prior research [39], the results of this study suggest that the establishment of social norms can affect individuals’ actions toward reducing food waste. It also supports that anti-wastage social norms not only stimulate individuals’ opposition to food waste [40,41] but also positively affect their intention to reduce food waste [18].

*“Usually when I throw food away, I tell myself that I should not waste food like this all the time, otherwise, I will have nothing to eat in my next life. I just feel guilty.”*

(B_24)

#### 4.1.4. Food Expenditure (13%)

Financial concerns have been identified as essential to the reduction of household food waste [6]. People who are more concerned about food expenditures are inclined to generate less food waste [23]. The interviews suggest that the respondents intend to eat up all available food since they feel that throwing away food is like throwing away money. This is similar to a finding by Revilla and Salet (2018) that economically aware households are willing to consume leftovers to save money even if avoiding food waste is not their priority [42]. However, this does not mean that household consumers prefer to buy low-priced food in order to reduce food expenses. Instead, interview results reveal that people are willing to buy high-quality products with slightly higher prices to increase the chances of the food being consumed by their family members. The small family respondents seemed to prefer purchasing slightly more expensive yet good-quality food over cheap poor-quality products. More specifically, they believe that purchasing poor-quality food causes more waste of money as these are least preferred by family members and are prone to decay. Conversely, most respondents expressed that they are changing their habit of buying food simply because of low price, choosing instead to purchase high-quality products in small packages that help to eliminate household food waste. This result is different from previous studies which indicated that households tend to buy too much food because of preferential prices [6,26].

*“If I cannot use that much, why not buy a small package? I would rather buy a small package even if it is slightly more expensive than a large package. It is seemingly cost-effective. When little is used, a large portion will be left and thrown away. Isn’t it money-wasting and food-wasting?”*

(B_23)

### 4.2. Barriers to Minimizing Food Waste

#### 4.2.1. Lack of Knowledge in Assessing Edibility (50%)

Blichfeldt, Mikkelsen, and Gram (2015) indicated that household consumers adopt two different strategies to assess the edibility of food, namely objectification and internalization. Customers adopting the objectification strategy judge the edibility of food by external standards (e.g., best-before dates), while those who are adopting internalization use their senses and knowledge to judge whether food is still edible [43]. The analytical results of this study revealed that most respondents adopt those two strategies alternately. In other words, they judge food edibility by both observing its external property and assessing the food using their knowledge. However, traditional markets in Taiwan do not have shelf-life labels for their food products, despite being the main channel for food purchases. Consequently, most respondents are compelled to resort to internalization to evaluate the edibility of their food purchases. While the internalization relies much on an individual’s sense and knowledge, this strategy also depends on an individual’s rule of thumb which may lack a scientific basis or objective judgment. Household consumers without sufficient knowledge or skill in assessing food may generate much food waste due to their risk perception and subjective judgment on food. Consistent with prior research [3,26], the results of the current study indicate that most interview respondents would rather throw away food than bear the risk of disease posed by eating leftovers and stale food. Additionally, the results found that people tend to think that leftovers have lower nutritional value and often taste staler than fresh food, and are thus not good for health. This finding indicates that food safety, nutritional value and taste are households’ main concerns influencing whether leftovers would be consumed or thrown away, in turn increasing the quantity of food waste.

*“If we think the food is not okay or not fresh, we will throw it away without hesitation.”*

(A_9)

*“If fresh food such as meat or fish has been stored in the refrigerator for too long with its appearance seeming normal but with weird look or smell, I will tell my family members to throw it away.”*

(B_26)

#### 4.2.2. Unexpected Food from Someone or Occasion (22%)

Many unexpected foods of households in Taiwan come from the region’s gift-giving culture. Gift-giving strengthens an interpersonal relationship, showing respect to a person’s status and serving as a tool for market exchange [13]. Taiwan has inherited gift-giving from the Chinese culture and takes gift-giving as one of the important channels to connect with others. Many Taiwanese festivals use special food to show respect and memorialize tradition and culture (e.g., eating Zongzi on the Dragon Boat Festival and mooncakes on the Mid-Autumn Festival). In these special traditional festivals, “food for occasion” has become the most popular gift-giving choice [12]. However, this food-giving tradition may cause hidden worries over food waste for small households. This is especially true when giving gifts involves wealth and status symbols, where givers often give much more food than the recipients can consume and thus leads to food waste problems [12]. Additionally, the interview results show that the gift food recipients tend to dispose of the food when they do not like it. As a respondent stated:

*“For example, my daughter’s mother-in-law ordered pitayas from the place of origin and gave me these fruits. She sent me two boxes, with each box containing more than 50 pitayas, I then had two boxes of more than 100 pitayas. How can I finish them all by myself?”*

(A_6)

*“It is not that the food we are given is not delicious, it is more that our family may not like it, or it is really not what we would eat as the food is overly sweet.”*

(B_25)

#### 4.2.3. Unexpected Dining Schedule (16%)

Unexpected dining schedules includes spontaneous decisions such as having take-outs, going out for dinner, or family members unable to return home for dinner on short notice. This study found that unexpected conditions prevent household food providers from planning and estimating the necessary food correctly, possibly resulting in cooking too much and decay of the prepared food. This result echoes previous studies [44,45], demonstrating that the unpredictable eating habits and schedules of family members make shopping plans difficult for people preparing food. Despite planning, unpredictable eating behaviors would still eventually lead to a food waste problem.

With the increased proportion of married women going out to work in Taiwan, the proportion of household expenditure on dining out has risen from 9% to 13% in the past decade. However, the household expenditure on food has not fallen significantly [34]. This is also consistent with prior studies [32,46], suggesting that households that often dine out do not spend less on household food purchases. Consequently, unexpected dining invitations and short notices in dining schedules lead to a large amount of thrown food at home.

*“Sometimes my husband has to work overtime at short notice, or something comes up, he or our children cannot come home for dinner, then the food bought or cooked may be wasted.”*

(A_12)

#### 4.2.4. Lack of Environmental Awareness (12%)

As kitchen waste in Taiwan being sent to pig and composting farms has exceeded the amount these could handle, the excess waste is treated by incineration. However, incinerating leftover food produces heavy metals and dioxins, which harm human health and damage the environment. According to the finding of this study, the majority of respondents believe that kitchen waste can be reused as feed for pigs or organic fertilizer for crops, not realizing that the subsequent treatment of kitchen waste poses a serious threat to the environment and human health. Some respondents even thought that as keeping excess and unwanted food in a refrigerator would increase economic and preservation costs, directly throwing them away is a better choice. Moreover, the respondents tend to underestimate the extent of their food waste in daily life since they are not concerned about their food waste behavior. This finding is similar to Neff et al. (2015), indicating that a majority of American consumers estimate their food waste as much lower than the amount indicated by national statistics [20]. Individuals who do not have environmental awareness consider many of their behaviors related to food waste as irrelevant to environmental damage. Consistent with previous studies [17,28], the interview findings show that a lack of environmental awareness leads to resistance to lowering household food waste.

*“Throwing food away does not really feel bad… To keep it in the refrigerator will waste power and taint other food with a bad smell. If nobody wants to eat it, then it should be thrown away.”*

(B_19)

*“I think the kitchen waste should be used to feed pigs or sent somewhere to bury till it rots, through which, it may produce limited hazards to the environment.”*

(A_15)

### 4.3. Food Waste Prevention Strategies

#### 4.3.1. Planned Purchase Schedule (33%)

Careful planning purchase schedules is an effective strategy to prevent over-purchases and food waste [47]. This study verified the recommended purchase planning strategies from previous studies, which include writing a shopping list, compiling meal plans in advance and checking inventories before shopping [17,19,22]. Additionally, the interview results indicate that Taiwanese couples increasingly share household expenditure and affairs due to the growing prevalence of the dual-income family structure. When either one of a couple purchases household food without communication in advance, they tend to buy the same food in larger quantities than they need, thereby increasing the risk of food waste. Therefore, increased communication among family members is an important strategy for pre-purchase planning. As Farr-Wharton, Foth, and Choi (2014) suggested, effective communication helps households to avoid purchasing the same food [26]. Alternatively, repeated food purchase can be avoided by designating a household food purchaser responsible for the family food purchase planning.

*“So my husband will buy… Sometimes his company will order some fruits or food together, but he usually won’t tell me and just bring them back. When I purchase the same food by accident, the doubled food becomes too much.”*

(A_10)

#### 4.3.2. Skills to Keep Food Fresh and Longer (29%)

Dobernig and Schanes (2019) stressed that the temperature of food preservation is significant for keeping food fresh [11]. Since Taiwan is located in the subtropical region, its climate is normally sultry and humid and is not good for food preservation. Another problem for food preservation is that many food items are sold in bags or boxes at the purchasing channels, while few are sold in “pieces”. Buying a large quantity of food makes it more difficult to preserve, particularly for small households. Specifically, the Taiwanese cooking style where dishes are prepared with small quantities of ingredients (e.g., soups are sprinkled with scallion or coriander) may result in overbuying and the remaining food items are eventually stale and thrown away. To resolve the problem, the majority of respondents cited freezing as the most common method used for food preservation [47]. For instance, food items such as rhizomes, leftover white rice, and decorative food materials such as green onion, garlic, and chili pepper can extend their fresh and edible period after cleaning, sub-packaging and freezing. In addition, the interview results show that the progress of science and technology has provided better environments for food preservation, such as a new type of refrigerator built with a vacuum interlayer to protect food from oxidation and extend the food’s shelf life, or a convenient vacuum packing machine, which has been recognized as an effective household appliance for food preservation in Taipei.

*“Rhizomes, such as potatoes or sweet potatoes, will sprout after being stored for a long time. I’m used to processing them first, washing them, cutting them into the required size, and then storing them in the freezer.”*

(A_14)

*“Some food can be stored in vacuum, including meat and seafood, such as anchovy larvae, shrimps and chicken, which can be stored in vacuum bags for a long time… Because food can be stored in vacuum bags for about half a year, it is safe as long as you finish it within the time limit.”*

(B_23)

#### 4.3.3. Understanding Family Preferences and Leftover Management (28%)

Previous literature has indicated that households with children tend to produce most food waste [21], partly because parents cannot predict their children’s eating behaviors and food preferences [20,25]. Similar to previous studies, the current study found that the interview respondents as household food providers would purchase and prepare food based on their family members’ food preferences. Ensuring that the food is cooked properly and meets the preferences of family members reduces the possibility of having leftovers. However, ensuring that no food is ever left from a meal is difficult, resulting in leftovers being frequently thrown away. In order to reduce food waste, strategies such as making reused leftovers acceptable to family members and leftover management have become important management skills for household food providers [25]. Interview results identified several leftover management strategies to eliminate household food waste. For example, most respondents stated that they would divide the large portion of dishes (e.g., stewed meat) into small packages for each meal and freeze them to avoid repeated heating before serving. Another strategy for leftover management is to reuse leftover food, such as by cooking leftover rice into porridge as breakfast for the next morning, or reusing leftovers in new dishes. This is consistent with Boccia et al.’s (2019) research, indicating that reuse strategies can be applied to tomato waste that can be converted into marketable products [48]. Therefore, reusing leftover food is an effective management strategy to reduce household food waste.

*“I know my children’s tastes and what they like. Basically, I will cook what they like and avoid what they don’t eat. I create new dishes containing their favorite ingredients.”*

(A_9)

*“For example, if the salmon is pan-fried and not finished at the end, I will take off the meat and turn it into salmon fried rice on the next day… Or the fish will be stored in the refrigerator for use in other dishes later.”*

(A_4)

#### 4.3.4. Sharing Additional Food and Co-Procurement and Cooking (10%)

Redistribution of additional food and food exchange between households are also alternative ways to reduce food waste [49]. Some respondents said that they often use food sharing platforms to reduce food waste. Many non-governmental organizations have recently emerged in Taipei, such as the surplus food community and food bank, which allows people to share additional food and facilitates co-procurement. The leftover food community uses social media to communicate with its members, creates a food sharing and exchanging platform that can help people match the food they give to someone who is in need so that the surplus food can be used timely and effectively.

*“If I have a large quantity of fruits, I will keep some of them for my family, for the remaining part I usually will ask the surplus food community if anyone wants the fruits for free.”*

(B_20)

Interview results reveal that co-procurement and co-cooking are also common among respondent households. Co-procurement includes purchasing large quantities together to be shared by relatives, friends or regional group shopping communities. This approach not only shares purchasing quantity but also allows households to enjoy preferential prices for large quantities. Co-cooking means cooking and sharing meals together with someone who lives nearby, and is mainly practiced by small-size households with four members or less. Most co-cooking occurs for dinner meals. In Taipei, for some double-income parents who cannot prepare dinner on time after work will cook and share dinner together with relatives or friends nearby (usually parents-in-law) to share food expenditure. This strategy not only releases time pressure for double-income small families in food preparation, but also solves the food waste problem caused by cooking excessive amounts of food.

*“For example, I always buy chicken in large quantities with my mother-in-law together from the same butcher shop. Before buying, I check how much chicken I need and then we will order and share the chicken together.”*

(A_11)

## 5. Conclusions

Few previous investigations have focused on household food waste in Chinese culture. This study explore relevant factors affecting the reduction of household food waste in Taiwan and identifying management strategies to prevent household food waste. Analytical results of this study help improve the understanding and application of household food waste reduction.

Interview results indicate that determinants for household food waste reduction mainly incorporate food knowledge and practices and external situational factors rather than the individual’s internal factors. Aside from internal factors of individuals that influence household food waste behavior (e.g., attitude, norms, and intention), situational (e.g., shopping environment) and food knowledge and practice factors (e.g., knowledge of food edibility) should also be given much more attention. Whereas prior research focused on the application of the TPB model, this study provides additional value by identifying knowledge and practices and situational factors as the main influence of household food waste behavior. Additionally, this study finds that social and cultural factors, such as gift-giving culture and social norms, have a considerable effect on household food waste in Taiwan. The results further deepen our understanding of the underlying causes of Chinese household food waste and remind us to pay close attention to the socio-cultural impacts in future studies. Based on the social practice theory, this study emphasizes that individual behaviors should be placed in a wider range of social, economic and cultural contexts of daily life. Food waste is not only an individual’s internal concern; it should also be studied in an external perspective where interactions among individuals and their daily food practices can provide insightful contribution.

Additionally, this study identifies several strategies to prevent household food waste and provides relevant practices for Taiwanese and Asian families. First, planning shopping schedules and designating household food purchasers are most important to avoid repeated purchasing. Second, household food providers need to enhance their knowledge and skills of food preservation. Most Asian countries have humid and sultry climates, where food is particularly vulnerable to decay without proper preservation. Hence, this study suggests that the application of new technology and knowledge on food preservation can help household food handlers to keep food fresh for longer. Moreover, food waste can be reduced by understanding the taste and food preferences of family members as well as adopting leftover management strategies to ensure that food is acceptable and consumed. Finally, household surplus food can be lowered by food sharing, co-procurement and co-cooking among families and communities. Sharing purchase quantities to reduce cost is an effective strategy to prevent food waste among double-income small households.

## 6. Limitations and Future Studies

This study still has some limitations. First, since this study was performed in Taiwan, its findings may not be generalizable to other countries, as different regions have their own values and culture connected with household food consumption and habits. Second, this study only interviewed households in Taipei, the metropolitan area of Taiwan. The inferences of the research results are more suitable for solving the household food waste problem in urban than rural areas. Future studies should focus on investigating household food waste behavior in rural areas or in different social and cultural backgrounds. Third, the present research was conducted by a qualitative approach. Future studies may consider using a mixed approach combining both qualitative and quantitative methods with a longitudinal data collection to further understand household food waste and effective management strategies for food waste reduction. Finally, this study consisted of 27 household food providers as in-depth interviewees, the limited size of the sample may be a limitation of the research. However, previous studies have selected similar or even much lower numbers of the sample than the current study in terms of the sample size of interviewees (e.g., Cappellini and Parsons, 2012; Graham-Rowe et al., 2014; Porpino et al., 2015). Future studies are encouraged to extend the sample size in order to incorporate wider range of viewpoints of Taiwanese households.

## Figures and Tables

**Table 1:** Factors and management strategies for household food waste.

Article	Theme	Method	Respondents	Factors	Management Strategies
[25]	Mealtime, leftovers, sacrifice and family membership	Ethnographic study	20 households living in UK. The age range was early 30 s to early 50 s.	Moral norm, unpredictable eating patterns and preferences, lack of acceptance of leftovers	Managing leftovers, proper and systematic storage practices
[6]	Motivations and barriers to minimizing household food waste	Qualitative study	15 UK household food purchasers. The age range was 21 to 75.	Waste concerns, doing the ‘right’ thing, a ‘good’ provider identity, minimizing inconvenience, exemption from responsibility	Food management
[19]	Food waste behaviors	The ‘lenses’ of different academic disciplines	Waste & Resources Action Programme (WRAP) (UK)	Attitude, norm, habit, emotion, awareness	Planning, shopping, storing, cooking knowledge
[17]	The importance of planning and shopping routines	Questionnaire survey	244 Romanian consumers. The mean age of the participants was 38 years, and 86% of respondents were female.	Moral attitudes, waste concerns, subjective norm, perceived behavioral control	Planning and shopping routines
[3]	Food waste causes and potential for action	Literature review and expert interviews	Consumer in the U.S. and the U.K.	Good provider identity, environmental awareness, doing the ‘right’ thing, lack of acceptance of leftovers	Planning of food shopping and meals, shopping list, proper and systematic storage practices, expiration date monitoring, assessing edibility, knowledge about shelf-life and how to extend it
[8]	Household food waste reduction	Questionnaire survey	279 UK residents. The mean age of the participants was 35 years, and 80% of respondents were female.	Attitude, subjective norm, perceived behavioral control, self-identity, anticipated regret, moral norm, descriptive norm	No mention
[18]	Determinants of consumer food waste behavior	Questionnaire survey	1062 Danish consumers. The mean age of the participants was 48 years, and 53% of respondents were female.	Injunctive and moral norms, attitudes towards food waste, perceived behavioral control	Planning of food shopping and meals, shopping list, shopping routines, portion control, managing leftovers
[21]	Motivators and barriers of self-reported amounts of food waste in households	Questionnaire Survey	796 Swiss-German residents. The mean age of the participants was 57 years, and 59% of respondents were female.	Attitude, subjective norm, personal norm, knowledge, household, planning habits, the good provider identity, perceived health risk	Food storage knowledge, knowledge about shelf-life and how to extend it
[30]	Household food waste	Literature review	Western countries	Awareness and attitudes, social norm, health perception, good provider identity, eating preferences, lifestyles	Planning, storing, portion control, managing leftovers, assessing edibility, knowledge about shelf-life and how to extend it
[33]	Household food waste	Questionnaire Survey	500 Greek households. The mean age of the participants was 36 years, and 60% of respondents were female.	Shopping habits, eating preferences	Proper and systematic storage practices, expiration date monitoring, portion control
[9]	Food waste behavior	A temporally lagged design	172 UK consumers. The median age of participants was in the range of 50–59, and 59% of respondents were female.	Subjective norm, attitude towards food waste, perceived behavioral control, habitual food waste behavior, negative emotion	No mention
[4]	Attitudes and behaviors shaping household food waste generation	Questionnaire Survey	1264 Lebanon households. 86% of respondents were female.	Awareness, eating-out in restaurants, buying special offers, moral norm	Managing leftovers
[22]	Household food waste practices and their policy implications	Systematic review	Europe	Environmental awareness, norms, attitudes, perceived behavioral control, health perception, good provider identity, eating preferences, minimizing inconvenience, time constraints; unplanned events, eating-out, inadequate communication between household members	Knowledge about planning, shopping, storing, and cooking

**Table 2:** Demographic information of the respondents.

Location	Code	Gender	Household Size	Age	Occupation	Education
Taipei City	A_01	F	3 (2 parents/1 child)	42	Office worker	University
	A_02	F	4 (2 parents/2 children)	34	Housewife	University
	A_03	M	6 (2 parents/1 child/1 grandmother/1 great-grandmother/1 servant)	33	Administration staff	Graduate school
	A_04	F	3 (2 parents/1 child)	35	Office worker	University
	A_05	M	3 (2 parents/1 child)	40	Administration staff	University
	A_06	F	7 (2 parents/1 nephew/2 grandparents/2 servants)	63	Company owner	College
	A_07	F	4 (2 parents/2 children)	34	Store owner	University
	A_08	F	3 (2 parents/1 child)	44	Bakery owner	College
	A_09	F	3 (2 parents/1 child)	32	Housewife	University
	A_10	F	4 (2 parents/2 children)	42	Housewife	University
	A_11	F	4 (2 parents/2 children)	40	Housewife	University
	A_12	F	4 (2 parents/2 children)	44	Housewife	College
	A_13	F	4 (2 parents/2 children)	38	Housewife	University
	A_14	F	3 (2 parents/1 child)	66	Housewife	College
	A_15	F	4 (2 parents/2 children)	68	Housewife	College
	A_16	F	4 (2 parents/1 child)	37	Office staff	University
	A_17	F	4 (2 parents/2 children)	32	Cook	College
New Taipei City	B_18	F	ordinary day: 2 (2 parents) holiday:14 (2 parents/5 children/7 grandchildren)	52	Cleaning lady	Vocational high school
	B_19	F	5 (2 parents/3 children)	44	Teacher	University
	B_20	F	3 (couple/1 mother-in-law)	42	Administration staff	University
	B_21	F	5 (2 parents/3 children)	50	Factory worker	Vocational high school
	B_22	F	3 (2 parents/1 child)	54	Administration staff	University
	B_23	F	6 (2 parents/1 son/1 daughter-in-law/2 grandchildren)	48	Secretary	High school
	B_24	F	5 (2 parents/3 children)	40	Administration staff	Graduate school
	B_25	F	4 (2 parents/2 children)	39	Office worker	Graduate school
	B_26	F	4 (2 parents/2 children)	43	Office worker	Graduate school
	B_27	F	3 (2 parents/1 child)	41	Administration staff	Graduate school

**Table 3:** Interview questions and their links to the extant literature.

Themes/Interview Questions	References
1. Food purchasing practices (e.g., How do you shop for food for your family? Can you describe a typical food shopping trip? How do you decide what food you are going to buy? How much do you spend on food in an average week? How often do you usually do your main shopping trip? How often do you usually do a smaller ‘‘top up’’ shopping trip?)	[6,7,11,17,22,30]
2. Food storage practices (e.g., Can you describe a typical food storage process after you shop? Can you share special preservation methods to reduce food spoilage?)	[7,11,22,30]
3. Food preparation and cooking (e.g., How do you decide how much food you cook each time? How do you plan the meals you cook every time? What is your attitude towards the leftovers? How do you deal with the leftovers?)	[7,11,22,30]
4. Food waste behaviors (e.g., Tell me about your thoughts and feelings regarding throwing food away. What caused you to throw away the food? Can you describe why you think this happened? How did you decide that food should be discarded? How much food do you throw away from what you buy in a regular week?)	[6,17,22,30]
5. Methods and strategies for the reduction of food waste (e.g., What are your food management practices? What are the most effective ways to avoid or reduce the amount of food thrown away? Are there any obstacles that will prevent you from reducing food waste? How will you deal with this problem?)	[6,17,22]
